# Neighborhood characteristics and violence behind closed doors: The spatial overlap of child maltreatment and intimate partner violence

**DOI:** 10.1371/journal.pone.0198684

**Published:** 2018-06-07

**Authors:** Enrique Gracia, Antonio López-Quílez, Miriam Marco, Marisol Lila

**Affiliations:** 1 Department of Social Psychology, University of Valencia, Valencia, Spain; 2 Department of Statistics and Operations Research, University of Valencia, Valencia, Spain; University of South Carolina, UNITED STATES

## Abstract

In this study, we analyze first whether there is a common spatial distribution of child maltreatment (CM) and intimate partner violence (IPV), and second, whether the risks of CM and IPV are influenced by the same neighborhood characteristics, and if these risks spatially overlap. To this end we used geocoded data of CM referrals (N = 588) and IPV incidents (N = 1450) in the city of Valencia (Spain). As neighborhood proxies, we used 552 census block groups. Neighborhood characteristics analyzed at the aggregated level (census block groups) were: Neighborhood concentrated disadvantage (neighborhood economic status, neighborhood education level, and policing activity), immigrant concentration, and residential instability. A Bayesian joint modeling approach was used to examine the spatial distribution of CM and IPV, and a Bayesian random-effects modeling approach was used to analyze the influence of neighborhood-level characteristics on small-area variations of CM and IPV risks. For CM, 98% of the total between-area variation in risk was captured by a shared spatial component, while for IPV the shared component was 77%. The risks of CM and IPV were higher in neighborhoods characterized by lower levels of economic status and education, and higher levels of policing activity, immigrant concentration, and residential instability. The correlation between the log relative risk of CM and IPV was .85. Most census block groups had either low or high risks in both outcomes (with only 10.5% of the areas with mismatched risks). These results show that certain neighborhood characteristics are associated with an increase in the risk of family violence, regardless of whether this violence is against children or against intimate partners. Identifying these high-risk areas can inform a more integrated community-level response to both types of family violence. Future research should consider a community-level approach to address both types of family violence, as opposed to individual-level intervention addressing each type of violence separately.

## Introduction

Child maltreatment (CM) and intimate partner violence (IPV) are both major social, public health, and human rights problems, highly prevalent globally, and with severe and far-reaching consequences not only for victims but also for the wider society [1–13]. CM and IPV are two forms of family violence (the umbrella concept under which these two types of violence among intimates are often included) with common characteristics and risk factors [14–17]. Both forms of violence are considered risk factors for the other [18–22], and as the high rates of co-occurrence of CM and IPV reported in the literature illustrates, they tend to overlap in the same families [15,16,23–25]. Although existing research has examined the co-occurrence of these two types of violence in the same families, no research has examined whether the risk of CM and IPV also overlap in the same neighborhoods. This is a relevant research question, because if the interconnection of CM and IPV also occurs at the community-level, neighborhood-level interventions targeting high-risk areas would emerge as a cost-effective and integrative public health approach to reduce both types of family violence within the same policy agenda.

CM and IPV have both been considered as types of crime that tend to occur ‘behind closed doors’ [26,27]. However, and despite the often-hidden nature of these offenses, a substantial body of research supports the idea that, beyond individual and relational factors, ‘place’ also matters for both CM and IPV. Research based on social disorganization and ecological perspectives points to the importance of community characteristics (e.g., neighborhood concentrated disadvantage) in explaining rates of both CM and IPV [28–32]. As similar neighborhood risk factors have been linked to these two types of family violence, it is likely that they will both tend to occur more often in neighborhoods that are characterized by those risk factors. In this study we hypothesize that the risk of CM and IPV will overlap spatially in the same neighborhoods.

Previous research has showed that CM and IPV, respectively, tend to spatially concentrate in certain city areas [33–39]; however, no studies have yet examined both types of family violence simultaneously, using appropriate spatial techniques to analyze whether the risks of CM and IPV are influenced by the same neighborhood characteristics, and tend to spatially overlap.

## Material and methods

### Outcome variables

The study was conducted in the city of Valencia (Spain). Valencia is the third largest city in Spain with a population of 736,580 (2013 data). For this study, census block groups were used as the neighborhood proxy, and were the unit of analysis. Valencia is divided into 552 census block groups (with populations ranging from 630 to 2,845).

Two different outcomes were collected for this study. First, addresses for all IPV cases with an associated protection order issued between 2011 and 2012 were provided by the Valencia Police Department. Protection orders represent severe cases of IPV, as they are issued by a court of law to provide special protection for the victim. In this study, we consider only male-against-female IPV. The number of protection orders in this period was 1,450. Second, addresses for all child maltreatment referrals investigated by the city’s Child Protection Services during the same years (2011 to 2012) were provided by this agency. To avoid data dependency, child maltreatment referrals were per family unit (i.e., each family investigated is included only once), as a family can have more than one child with protection measures. This did not apply to IPV protection orders, as only one protection order was associated with each case. The total number of family units with child maltreatment referrals was 588. Data for IPV cases and CM referrals were geocoded using the address where the incidents occurred.

This research was conducted under two Joint Research Agreements signed between the University of Valencia and the Valencia Police Department, and the Social Welfare Department of the Valencia City Hall, respectively. Both agencies, the Valencia Police Department, and the Valencia Social Welfare Department through its Child Protection Services, participated actively in this research project by facilitating the data required. Permissions to access police records regarding address of IPV incidents were granted by the Head of the Valencia Police Department. This research was approved and funded by the Spanish Institute for Women (Instituto de la Mujer, Ministerio de Sanidad, Servicios Sociales e Igualdad) and the European Social Fund as part of project MUJER2012-PI-154, and by the Spanish Ministry of Economy and Competitiveness as part of project PSI2014-54561-P. For this observational study, both the Ethics and Data Protection Committees of the University of Valencia were consulted to address potential confidentiality issues. All data used for this study was completely anonymized, and did not include any identifying information about individuals or families. Also for further anonymization, for analyses, all geographical coordinates corresponding to cases of IPV and CM were aggregated at the census block group level, so no individual addresses can be identified.

### Covariates

Different neighborhood-level characteristics were used as covariates based on a classic social disorganization theory approach [28,29,30,32,37]. We used three indicators to assess concentrated neighborhood disadvantage (neighborhood economic status, neighborhood education level, and policing activity, as a proxy of neighborhood public disorder and crime) one indicator of ethnic heterogeneity (immigrant concentration), and an indicator of residential instability.

*Economic status*: A factor analysis derived scale was used to measure neighborhood-level economic status; the scale contained 4 indicators: cadastral property value, percentage of high-end cars, percentage of financial business, and percentage of commercial business.

*Education level*: The value of this covariate was calculated as the average level of education in each census block group based on the percentage of the population in each education level category measured on a 4-point scale where 1 = less than primary education, 2 = primary education, 3 = secondary education, 4 = college education.

*Policing activity*: An index for each census block group was provided by senior police officers composed of 5 items measured on a 5-point Likert scale (0 = very low level of interventions, and 4 = very high level of interventions), which included police interventions such as drug-related crime, drunkenness and fights, vandalism, homeless people and truancy.

*Immigrant concentration*: Percentage of immigrant population in each census block group.

*Residential instability*: Proportion of the population who had moved into or out of each census block group during the previous year (rate per 1,000 inhabitants).

Table 1 summarizes the descriptive statistics for all variables.

**Table 1:** Variables (mean, standard deviation, minimum and maximum values) at the census block group level.

Variable	Mean (SD)	Min	Max
**Economic Status**			
Property value (€/m^2^)	260.10 (74.61)	111.50	590.70
High-end cars (%)	5.75 (3.62)	1.30	24.80
Financial activities (%)	18.15 (7.77)	0	43.20
Commercial activities (%)	34.03 (9.21)	7.50	66.40
**Education level**	3.15 (.33)	2.39	3.86
**Policing activity**	7.16 (3.99)	0	19
**Immigrant concentration (%)**	13.45 (6.53)	1.90	40.20
**Residential instability**	288.00 (87.98)	91.10	649.80

Abbreviations: SD, standard deviation; Min, minimum; Max, maximum €/ m^2^, euros per square meter.

### Statistical analysis

Two different analytic approaches were used. First, a Bayesian joint modeling analysis was conducted to examine the spatial distribution of IPV and CM cases [40]. We assumed that the outcomes followed a conditional independent Poisson distribution, and a shared component was introduced in the model:

$$Y_{ik}\sim Po(\mu_{ik})$$

$$\text{log}\;\mu_{i1}=\text{log}\;E_{i1}+\alpha_{1}+\phi_{i}*\delta +\psi_{i1}$$

$$\text{log}\;\mu_{i2}=\text{log}\;E_{i2}+\alpha_{2}+\phi_{i}/\delta +\psi_{i2}$$

where *Y*_*ik*_ are the observed counts for the outcome *k* (1 for IPV, and 2 for CM cases) in census block group *i*, μ_*ik*_ is the unknown mean, *E*_*ik*_ are the expected counts for the outcome *k* in *i*-census block group (in proportion to the number of female population over 16 years old for IPV, and in proportion to the number of family units for CM); *α*_*k*_ is the intercept, *δ* represents the scaling factor which allows the risk gradient for the shared component to be different for each outcome; *ϕ* is the shared component, and *ψ*_*i*1_ and *ψ*_*i*2_ are the two specific components. *ϕ* and *ψ* were composed of unstructured and structured spatial components [41]. We used the logarithmic transformation of the shared component proposed by Knorr-Held and Best [40]. The unstructured term was modeled by means of independent identically distributed Gaussian random variables, and the spatially structured term was modeled as a conditional spatial autoregressive (CAR) model [41]. Additionally, an improper uniform distribution was used for *α*_1_ and *α*_2_. We obtained the proportion of shared variance for each outcome (*η*_*k*_).

Second, after examining the common spatial distribution of IPV and CM, a Bayesian Poisson spatial regression modeling was conducted for each outcome. The five variables (economic status, education level, policing activity, immigrant concentration, and residential instability) were introduced in the models, and two spatial effects were assessed (structured and unstructured terms). The models were defined as follows:

$$\text{log}\;\mu_{ik}=\text{log}\;E_{ik}+\alpha +X_{i}\beta +S_{ik}+U_{ik}$$

where ∝ is the intercept, *β* represents the regression coefficients vector, *X* is the matrix of covariates, and *S* and *U* are the structured and unstructured terms, respectively. Thus, the log relative risk was modeled as *α* + *X*_*i*_*β* + *S*_*ik*_ + *U*_*ik*_.

Vague Gaussian distributions were used for the fixed effects *β*, while *α* was considered as an improper uniform distribution. *U* was modeled by means of independent identically distributed Gaussian random variables, and *S* was modeled as a CAR model [41].

Markov Chain Monte Carlo (MCMC) simulation techniques were applied to perform the Bayesian models [42], using software R and the WinBUGS package. 100,000 iterations were generated in each of the models assessed, and the first 10,000 were discarded as a burn-in period. The $\hat{R}$ parameter (the convergence diagnosis) showed a suitable convergence for all parameters.

## Results

**Fig 1:**
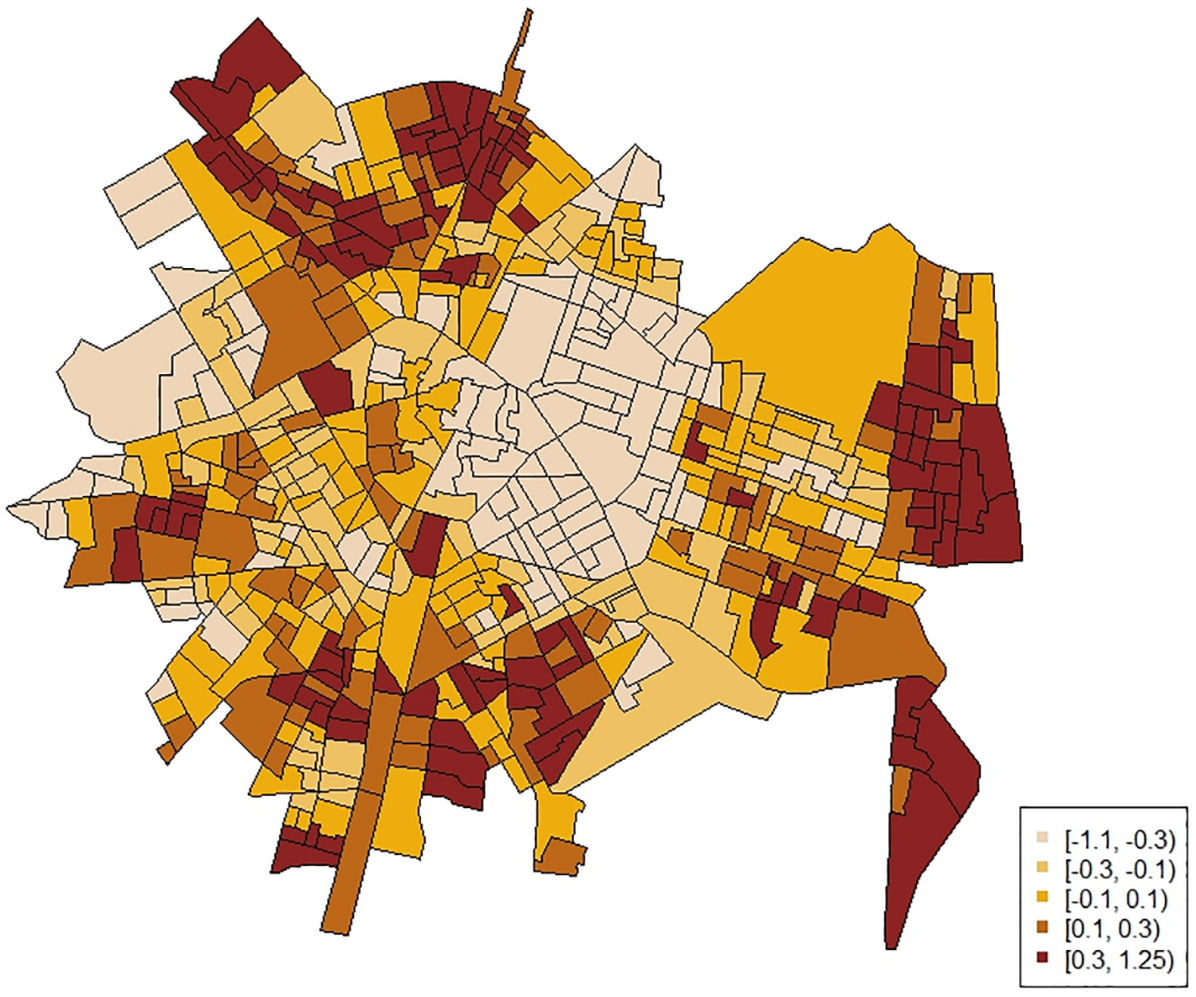
Shared spatial component from the joint modeling between child maltreatment and intimate partner violence risks.

Joint modeling results were firstly assessed (Table 2). For IPV cases, about 77% of the total between-area variation in risk was captured by the shared component. For CM referrals, about 98% of the total between-area variation in risk was captured by the shared component. Both outcomes, therefore, showed a common spatial pattern. Fig 1 illustrates this shared spatial component.

**Table 2:** Results from Bayesian joint modeling of the shared spatial component between intimate partner violence and child maltreatment risks.

	Mean	SD	CrI 95%
*α*_1_	-.096	.040	-.174, -.018
*α*_2_	-1.828	.082	-1.973, -1.670
*δ*	.703	.052	.606, .813
*η*_1_	.768	.162	.459, .995
*η*_2_	.978	.046	.832, .999

Abbreviations: SD, standard deviation, CrI, credible interval.

^1^ Intimate partner violence.

^2^ Child maltreatment.

Secondly, a Bayesian Poisson spatial regression was conducted for each outcome. In both models, the covariates presented the same relationship with the outcome (see Table 3). Specifically, results indicate that IPV and CM risks were higher in disadvantage neighborhoods, with lower levels of economic status and education, and higher levels of policing activity, immigrant concentration, and residential instability.

**Table 3:** Results from Bayesian Poisson spatial regression models of intimate partner violence and child maltreatment risks.

	Intimate partner violence			Child maltreatment		
	Mean	SD	95% CrI	Mean	SD	95% CrI
Intercept	.182	.617	-.982, 1.403	2.498	1.127	.222, 4.586
Economic status	-.131	.070	-.263, .004	-.145	.135	-.414, .117
Education level	-.287	.184	-.644, .060	-1.121	.341	-1.760, -.418
Policing activity	.016	.009	-.001, .033	.032	.015	.004, .060
Immigrant concentration	.030	.008	.013, .146	.018	.014	-.009, .044
Residential instability	.001	.009	-.001, .002	.001	.001	-.001, .003

Abbreviations: SD, standard deviation, CrI, credible interval.

**Fig 2:**
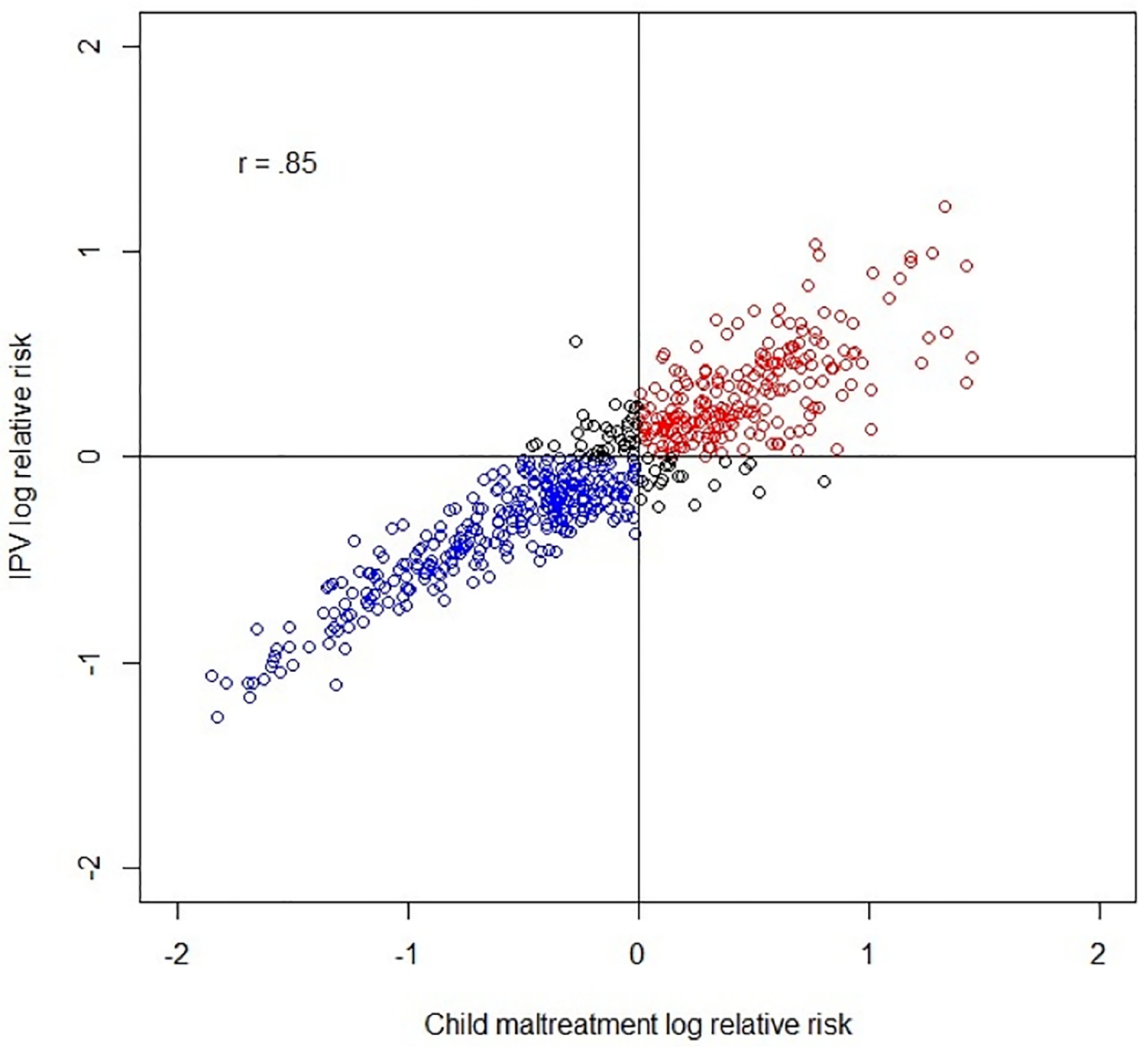
Scatter plot of the correlation between child maltreatment and intimate partner violence log relative risks.

**Fig 3:**
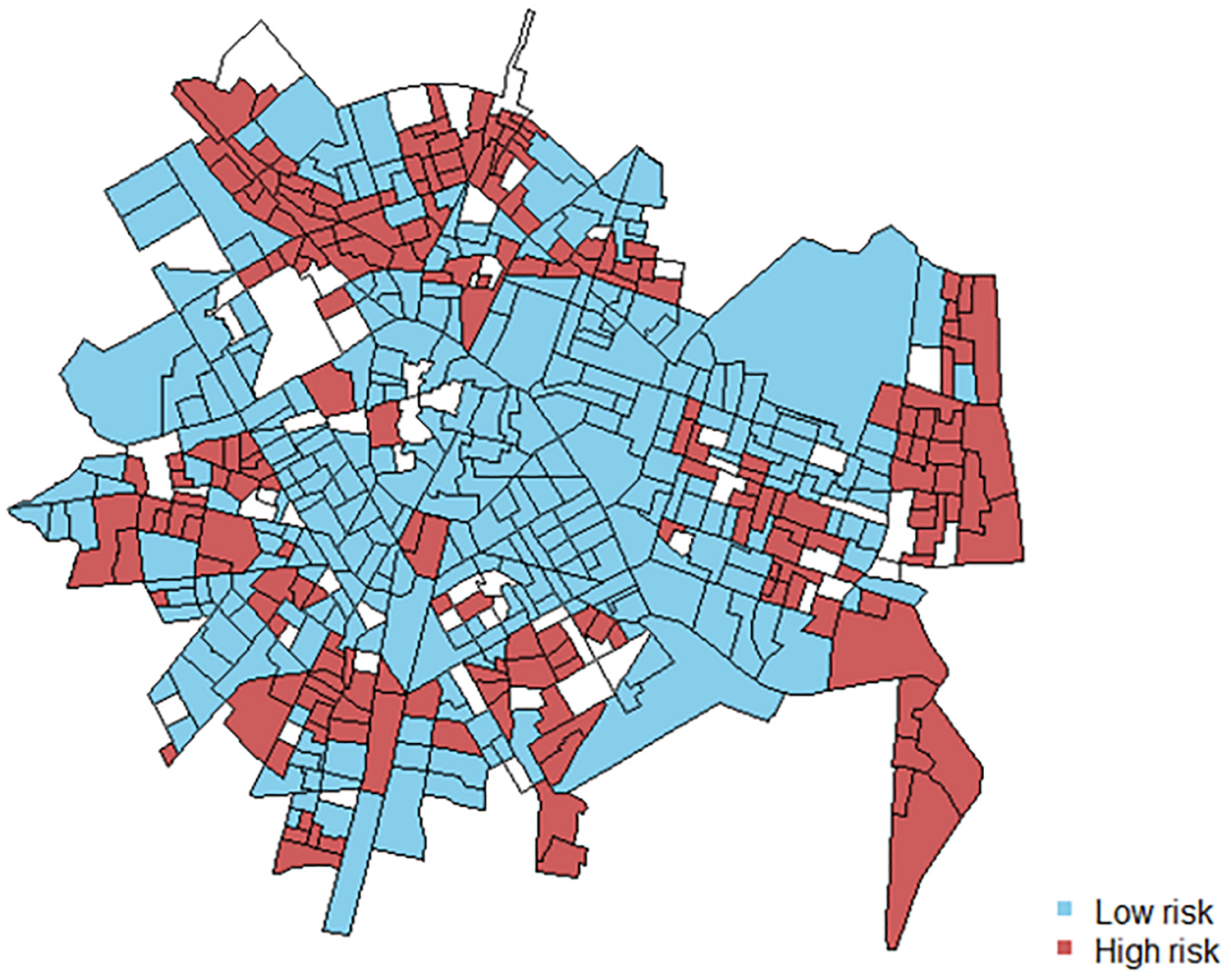
Map of the census block group with coincident low (blue) and high (red) relative risks for child maltreatment and intimate partner violence.

The relative risks of each model were correlated using Pearson’s correlation coefficient and a scatter plot. Fig 2 shows a high correlation between the log relative risks for IPV and CM (r = .85, CrI = [.81, .88]). In addition, Fig 3 displays the census block groups where the log relative risks for each outcome overlap (above-average and below-average risk levels). Most of the census block groups have low or high risks in both outcomes: only 10.5% of the areas have mismatched risks.

## Discussion

In this study, we analyzed first whether there was a common spatial distribution of IPV and CM in the city of Valencia and, second, whether the risks of IPV and CM were influenced by the same neighborhood characteristics, and if these risks spatially overlap. As hypothesized, results showed a common spatial distribution of CM and IPV, as a large percentage of the variation in both types of family violence across city areas was explained by a common spatial component (98% of the between-area variation for CM, and 77% for IPV). Results also showed that the same neighborhood characteristics (i.e., neighborhood economic status, neighborhood education status, policing activity, immigrant concentration, and residential instability) explained the risk of CM and IPV, and that these risks were higher in city areas with low economic and education status and with high levels of policing activity, immigrant concentration, and residential instability. Finally, our study clearly illustrated the spatial overlap of CM and IPV risks, as the correlation of the relative risks for the two types of family violence was .85, with only 10.5% of the city areas having mismatched risks (most areas of the city had coincident lower or higher risks than the city average in both outcomes).

The co-occurrence of CM and IPV in the same families is a well-established finding in the literature [15,16, 23–25]. What the present study highlights is that the overlap of these two types of family violence occurs not only at the individual (e.g., having been victim of CM and perpetrator of IPV later in life) or family levels (families in which both CM and IPV occur), but that the overlap between CM and IPV also occurs at the neighborhood level. In this regard, our results extend the common ground connecting these two types of family violence to the social context in which the families live, acknowledging the importance of neighborhood characteristics as common risk factors for both CM and IPV. This study not only supports previous research showing that ‘place’ matters for CM and IPV [28–32], independently, but provides evidence that the risks for both types of violence are simultaneously high or low in the same places. Our study illustrated that certain neighborhood characteristics indicative of social disorganization (i.e., low economic and education status, high levels of policing activity indicative of public disorder and high criminality, immigrant concentration, and residential instability) increases the risk of family violence, regardless of whether this violence is against children or against intimate partners.

Various psychosocial processes can be called upon to explain why neighborhoods where these characteristics concentrate are associated with family violence, increasing the risk of both CM and IPV [27–32]. First, from a social disorganization perspective, neighborhood concentrated disadvantage has been linked with reduced social control, and this diminished social control would be responsible for the relationship between these neighborhood characteristics and family violence. In disadvantaged neighborhoods, mistrust and a lack of social cohesion among residents may inhibit prosocial behavior and social control, reducing willingness to become involved in other residents’ lives (e.g., challenging other residents’ behavior toward their children or partners, or reporting known cases of CM or IPV), thus explaining the link between neighborhood disadvantage and family violence [43–50]. Second, isolation from mainstream values of what is acceptable in intimate relationships may also explain the higher risk of family violence in disadvantaged neighborhoods. Some behaviors involving the use of violence in intimate partner and parent-child relationships may be more tolerated and accepted in these neighborhoods, compared to mainstream norms or values regarding family violence (e.g., not disapproving of violent behaviors toward intimates in certain circumstances, or approving violence as an accepted way of settling family conflicts). These social norms have been defined as “cognitive landscapes or ecologically structured norms (normative ecologies) regarding appropriate standards and expectations of conduct” ([51] p. 63) that would provide the bases for a social climate of greater tolerance of family violence, whereby violence among intimates is not recognized or condemned as deviant but considered as a tolerated and accepted strategy that in these contexts, increases the risk of CM and IPV. From this perspective, disadvantaged neighborhoods can become fertile grounds for socialization that fosters attitudes accepting violence in intimate relationships, and internalizing these attitudes as acceptable violence becomes an appropriate strategy to resolve relationship conflicts, and either CM or IPV are not considered as important social problems deserving the mobilization of informal social control [44,52–58]. Finally, another possible explanation of the link between concentrated neighborhood disadvantage and risk of family violence is that these residential/social contexts can be highly stressful, reducing the quality of family life, and triggering violence in both parent-child and intimate partner relationships [27,30,32,59–62]. However, these variables were not available for this study, and we cannot test hypotheses on these alternative or complementary explanations.

This study also has several implications for advancing our understanding of and responses to family violence. Calls have been made for a greater integration of research addressing CM and IPV [15–17,63–68]. The interconnection between CM and IPV at the community level illustrated in this study not only advances our understanding of the causes of family violence by identifying common risk/protective factors at the community level, but also supports the need for a more integrative and broader approach in the prevention of family violence. Neighborhood conditions linked to both CM and IPV are modifiable risk factors, and identifying high-risk areas for both of them can potentially have an important preventive effect by targeting these two types of family violence within a same preventative/policy agenda. The high-resolution approach used in this study provides information that is more significant for policy relevance, as area-specific risk estimations are provided to inform a more localized intervention strategy. Furthermore, this community-level approach to address both types of family violence, as opposed to individual-level intervention addressing each type of violence separately, can reach a larger number of families in a more integrative and cost-effective way [28,31,65–71].

Finally, this study has both strengths and limitations. Examining for the first time the spatial overlap of CM and IPV within the same research framework, using appropriate analytical techniques and high-resolution disease mapping methods, thus providing greater policy relevance, are clearly among the study’s strengths. As for its limitations, this study uses only official cases of CM and IPV, and we cannot generalize our results regarding the overlap of CM and IPV to underreported cases, which is a common issue in both types of family violence [12,13]. Regarding the covariates used in this study, other socioeconomic measures such as rates of unemployment or income, other neighborhood variables linked in other studies to both types of family violence, such as alcohol outlets, and neighborhood processes such as those mentioned above were not available for this study [33,34,72–76]. Finally, the results correspond to a European city, and future research should examine the overlap of CM and IPV in other cities that may differ in structure and organization, as well as in other cultural contexts.

## Supporting information

S1 Data (CSV)
